# Long-Term Clinical Results of MR-Guided Stereotactic Body Radiotherapy of Liver Metastases

**DOI:** 10.3390/cancers15102786

**Published:** 2023-05-17

**Authors:** Fabian Weykamp, Philipp Hoegen, Sebastian Regnery, Efthimios Katsigiannopulos, C. Katharina Renkamp, Kristin Lang, Laila König, Elisabetta Sandrini, Eva Meixner, Carolin Rippke, Carolin Buchele, Jakob Liermann, Jürgen Debus, Sebastian Klüter, Juliane Hörner-Rieber

**Affiliations:** 1 Department of Radiation Oncology, Heidelberg University Hospital, 69120 Heidelberg, Germanysebastian.regnery@med.uni-heidelberg.de (S.R.); juliane.hoerner-rieber@med.uni-heidelberg.de (J.H.-R.); 2 Heidelberg Institute of Radiation Oncology (HIRO), 69120 Heidelberg, Germany; 3 National Center for Tumor Diseases (NCT), 69120 Heidelberg, Germany; 4 Clinical Cooperation Unit Radiation Oncology, German Cancer Research Center (DKFZ), 69120 Heidelberg, Germany; 5 Heidelberg Ion-Beam Therapy Center (HIT), Department of Radiation Oncology, Heidelberg University Hospital, 69120 Heidelberg, Germany; 6 German Cancer Consortium (DKTK), Partner Side, 69120 Heidelberg, Germany

**Keywords:** local control, adaptive radiotherapy, stereotactic body radiotherapy (SBRT), stereotactic ablative body radiotherapy (SABR), liver metastases, MR-guidance, biologically effective dose

## Abstract

**Simple Summary:**

Stereotactic body radiotherapy (SBRT) using direct magnetic resonance (MR) guidance is a comparably new technology and enables ablative treatment of liver metastases without invasive placement of fiducials. We aimed to evaluate long-term clinical outcome. Forty patients were treated for a total of 54 liver metastases (56% with online plan adaptation based on the daily anatomy). The most prevalent fractionation scheme was 50 Gy in five fractions. Estimated local control of the irradiated liver metastases was 75% at 2 years and overall survival was 57% at 2 years. We report the largest patient cohort to date, demonstrating promising long-term clinical results for SBRT of liver metastases applying MR-guidance.

**Abstract:**

(1) Background: Magnetic-resonance (MR)-guided stereotactic body radiotherapy (SBRT) allows for ablative, non-invasive treatment of liver metastases. However, long-term clinical outcome data are missing. (2) Methods: Patients received MR-guided SBRT with a MRIdian Linac between January 2019 and October 2021 and were part of an ongoing prospective observational registry. Local hepatic control (LHC), distant hepatic control (DHC), progression free survival (PFS) and overall survival (OS) were estimated with the Kaplan–Meier method. Toxicity was documented according to CTCAE (v.5.0). (3) Results: Forty patients were treated for a total of 54 liver metastases (56% with online plan adaptation). Median prescribed dose was 50 Gy in five fractions equal to a biologically effective dose (BED) (alpha/beta = 10 Gy) of 100 Gy. At 1 and 2 years, LHC was 98% and 75%, DHC was 34% and 15%, PFS was 21% and 5% and OS was 83% and 57%. Two-year LHC was higher in case of BED > 100 Gy (100% vs. 57%; log-rank *p* = 0.04). Acute grade 1 and 2 toxicity (mostly nausea) occurred in 26% and 7% of the patients, with no grade ≥ 3 event. (4) Conclusions: To our knowledge, this is the largest cohort of MR-guided liver SBRT. Long-term local control was promising and underscores the aim of achieving >100 Gy BED. Nonetheless, distant tumor control remains challenging.

## 1. Introduction

Stereotactic body radiotherapy (SBRT) allows for ablative local therapy of oligometastases in the liver [1,2,3,4]. Deployment of high irradiation doses increases local tumor control [5]; nonetheless, surrounding organs at risk (OARs) often hinder the application of tumoricidal doses at all [6,7,8,9]. Due to the low tissue contrast of conventional cone-beam CT scans (CBCT), liver metastases are especially difficult to treat with SBRT [10]. Moreover, the abdomen is subject to respiratory motion [11,12,13,14]. To account for respiratory motion, some CBCT-based SBRT approaches perform irradiation of the complete anticipated tumor motion track (internal target volume concept), which potentially increases the total amount of irradiated liver tissue [15]. Other approaches, e.g., the Cyberknife System, require invasively implanted fiducials to allow for tracking via continuous X-ray imaging [16]. Lately, magnetic-resonance (MR)-guided radiotherapy has become increasingly available, which provides high tissue contrast to distinguish the tumor volume from radiosensitive OARs. MR-Linac systems further allow for live MR-monitoring during the respective treatment sessions. Irradiation can then be applied through gating [17]. Moreover, online plan adaptation allows for treatment plans that are tailored to the daily anatomical situation in order to increase target coverage and reduce doses in surrounding OARs [18,19,20,21]. Clinical results of MR-guided SBRT are growing, but are still scarce and often have short median follow-up periods [20,22,23,24]. We sought to evaluate the long-term clinical benefit of MR-guided SBRT of liver metastases.

## 2. Materials and Methods

We report a subgroup analysis of a prospective observational registry comprising cancer patients with liver metastases who were deemed inoperable or refused surgery. The MR-Linac observational study was approved by the Ethics committee of the University Hospital Heidelberg (S-862/2019 and S-543/2018). Patients were treated with SBRT with an MRIdian Linac (ViewRay Inc., Mountain View, CA, USA; 0.345 T MRI scanner, 6 MV step-and-shoot intensity modulated radiotherapy) at the Department of Radiation Oncology at Heidelberg University Hospital between January 2019 and October 2021. Based on the statement by the “Stereotactic Radiotherapy” working group of the German Society of Radiation Oncology (DEGRO), single fraction doses ≥ 4 Gy and number of fractions ≤12 were defined as SBRT [25]. Early clinical results after a median follow-up of 9 months in 18 patients (45%) of this subgroup have been published before [26]. However, the mentioned analysis primarily focused on feasibility. Furthermore, a detailed dosimetric evaluation of the 30 liver metastases that received online adaptive treatment has been performed and published before and, hence, is not included in this work [27].

### 2.1. Treatment Characteristics

A thorough technical description of our MR-Linac system has been published earlier [28]. During treatment simulation with the MR-Linac, not only MR images were obtained, but also the patients’ compliance was re-evaluated regarding the small bore of the MR-Linac and the breathing commands. Three-dimensional (3D) TrueFISP images (acquisition time of 17 s to 25 s; 1.5 × 1.5 mm^2^ or 1.6 × 1.6 mm^2^; slice thicknesses 1.5–3 mm with varying fields-of-view) were obtained in deep inspiration breath-hold. Planar cine-MRI images in the sagittal plane addressed target motion [29]. No MR contrast fluid was used with the MR-Linac. After simulation with the MR-Linac, a planning CT scan with and without contrast fluid was performed for obtaining electron density information. The gross tumor volume (GTV) was delineated as the macroscopic tumor volume directly on the acquired MR-images during simulation as well as on all co-registered imaging modalities from the planning process. Clinical target volume (CTV) and planning target volume (PTV) margins were 5 mm and 3 mm.

During treatment, daily MR-guidance and breath-hold was carried out using the identical settings as during simulation with the MR-Linac. The newly obtained 3D MRI images were rigidly co-registered to the initial planning MRI with the GTV contours. OAR contours and the planning CT images were deformably registered to these new MRIs. The radiation therapists then delineated the OARs within 1 cm in craniocaudal and 3 cm in circumferential direction from the PTV (i.e., PTVexpand) [30]. Afterwards, the physician adapted the registered GTV and re-evaluated the OAR contours. The initial baseline plan was then recalculated using these modified structures to create the predicted plan. In case of PTV or OAR constraint violations, an adapted plan was generated by re-optimizing the predicted plan. Afterwards, the physicist performed on-table quality assurance. Plan adaptation was clinically implemented in our department in February 2020.

If visible on the daily TrueFISP sequence, the liver metastasis itself was used as the gating structure (region of interest; ROI). If not, a surrogate structure in close vicinity was chosen (e.g., large vessels or the surface of the respective liver segment). Isotropic expansion of the ROI by 3 mm was used to create the gating boundary. The irradiation was automatically paused if the gating structure left the gating boundary, including a 3% tolerance.

All treatment plans were aimed to fulfill complete conformal PTV coverage of at least 95% of the prescribed dose (V100% ≥ 95%), with a maximum dose (Dmax) of 125–150%. Whenever possible, three fractions of 15 Gy were prescribed (Dmax 150%). However, lesions larger than 5 cm were restricted to eight fractions of 7.5 Gy or five fractions of 10 Gy (each Dmax 125%). Ten fractions of 5 Gy (Dmax 125%) were used if the target lesion was adjacent to abdominal OAR. In two cases, homogeneous prescription was chosen for non-adaptive SBRT due to direct contact of the liver metastasis with surrounding OARs (Dmax 107%).

Table 1 shows the respective dose constraints for each fractionation scheme.

### 2.2. Statistical Methods, Response Evaluation and Follow-Up

Local hepatic control (LHC) was defined as local control of the irradiated liver metastases, whereas distant hepatic control (DHC) described the absence of newly detectable liver metastases, outside the PTV. Progression free survival (PFS) included tumor progression in any organ or any cause of death. Overall survival (OS) included any cause of death. LHC, DHC, PFS and OS were estimated using the Kaplan–Meier method, starting at the first day of the SBRT. Univariate analysis was performed through log-rank test. All statistical analyses were performed with SPSS software (IBM SPSS Version 24.0). A *p*-value of <0.05 was defined as significant. Evaluation of tumor response based on the Response Evaluation Criteria in Solid Tumors (RECIST 1.1). Toxicity during treatment and at first follow-up were documented according to the Common Terminology Criteria for Adverse Events (CTCAE v. 5.0). Follow-up examinations including a contrast-enhanced MRI or CT scan of the liver were performed six to eight weeks after SBRT as well as clinical examination. Afterwards, follow-up was performed every three months, but was not part of the prospective registry.

## 3. Results

Forty patients were treated with MR-guided SBRT for a total of 54 liver metastases between January 2019 and October 2021. Median age was 62 years and median Karnofsky performance score was 90% (Table 2). Most patients had systemic therapy 4 weeks before (68%) and after liver SBRT (40%). The majority of patients already had metastatic disease before diagnosis of the respective liver metastases, which were designated for SBRT (73%).

Thirty of the fifty-four lesions were treated with online plan adaptation (56%). The main underlying primary tumors of the 54 lesions were colorectal carcinoma (39%) and breast cancer (19%) (Table 3). The most common treatment scheme was 10 Gy in five fractions, leading to a median biological effective dose (BED) of 100 Gy (a/b = 10 Gy). Grades one and two toxicity were reported in 14 (26%) and 4 (7%) of the cases. Grade one events were nausea (n = 10), pain (n = 3) and dizziness (n = 1). Grade two events were nausea (n = 2), emesis (n = 1) and diarrhea (n = 1). No grade three or higher event occurred.

**Figure 1:**
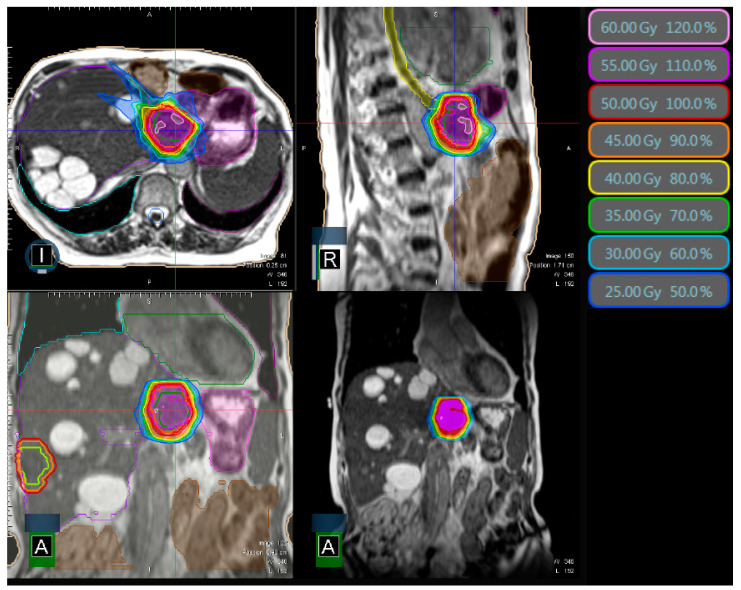
Typical MR-Linac treatment plan with colored isodose lines (50 Gy in 5 fractions) and surrounding small intestine/stomach (pink) and esophagus (yellow). The second liver metastasis in the contralateral liver lobe is shown delineated, which was irradiated concurrently in a different treatment plan. The patient has several known liver cysts. I = inferior, R = right, A = anterior perspective.

A typical treatment plan is depicted in Figure 1.

**Figure 2:**
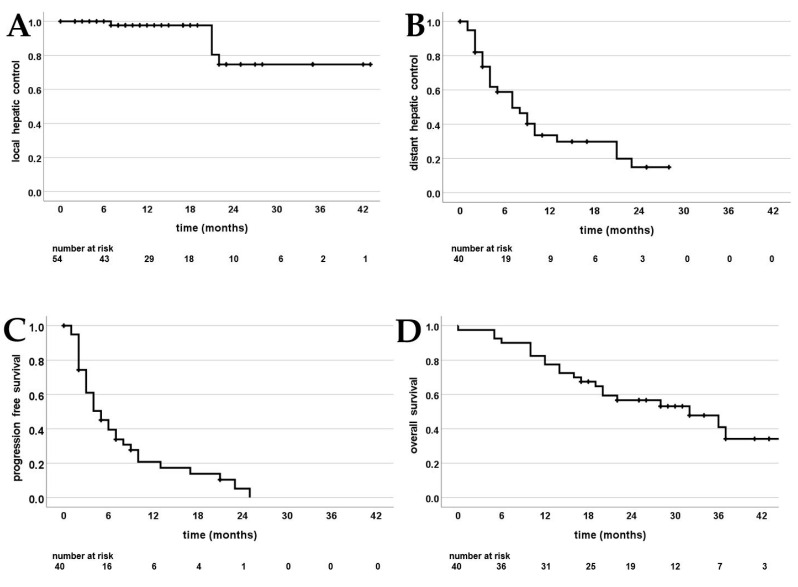
Kaplan–Meier curves; (**A**) local intrahepatic control of the irradiated liver metastases; (**B**) distant hepatic control (absence of newly developed liver metastases); (**C**) progression free survival; (**D**) overall survival.

Median follow-up was 22 months (0–45 months). One patient died immediately after the last fraction of SBRT from pneumonia, which was declared not associated with the liver SBRT. With 98% and 75% after 1 and 2 years, LHC was high (Figure 2A). Four out of forty patients had local recurrence within the PTV. The first of these patients received 50 Gy in 10 fractions, recurred after 7 months and had melanoma as the primary tumor. The second patient received 50 Gy in 10 fractions, recurred after 22 months and had pancreatic cancer as the primary tumor. The third patient received 50 Gy in five fractions, recurred after 22 months and had breast cancer as the primary tumor. Finally, the fourth patient received 50 Gy in five fractions for both liver metastases, which simultaneously recurred after 21 months with colorectal carcinoma as the underlying primary tumor. Most patients had systemic progression aside from the irradiated liver metastases: At 1 and 2 years, DHC was 34% and 15% and PFS was 21% and 5% (Figure 2B,C). Twenty-one patients (53%) died during follow-up, leading to an estimated OS of 83% and 57% at 1 and 2 years (Figure 2D).

**Figure 3:**
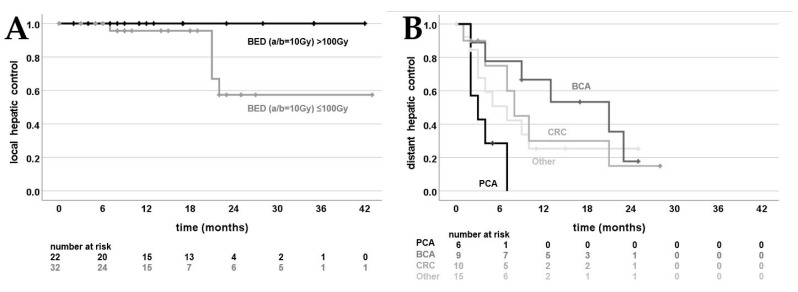
Kaplan–Meier curves; (**A**) local intrahepatic control of the irradiated liver metastases divided by biologically effective dose (BED; a/b = 10 Gy) 100 Gy (*p* = 0.04); (**B**) distant hepatic control (absence of newly developed liver metastases) divided by primary tumor: pancreatic cancer (PCA), breast cancer (BCA), colorectal carcinoma (CRC) and other (*p* = 0.04).

Univariate analysis with the factors from Table 2 and Table 3 in terms of each LHC, DHC, PFS and OS revealed two significant factors. A BED (a/b = 10 Gy) above 100 Gy was prognostic for local recurrence (Figure 3A): Estimated 2-year LHC was 57% for ≤100 Gy and 100% for >100 Gy (*p* = 0.04). Moreover, the primary tumor was associated with significantly different time periods for DHC (Figure 3B).

## 4. Discussion

Our long-term clinical results fall in line with previously published first results. Local control of 26 patients with MR-guided SBRT of liver lesions was higher in the study by Rosenberg et al. (80% vs. 75% in our study) [24]. However, the patient population was heterogeneous, with six patients treated for hepatocellular carcinoma and two for a primary cholangiocarcinoma. Another study by Rogowski et al. stated a local control rate of 100% [20]. However, median follow-up was short (5 months). Recently, a French retrospective study described an estimated local control of 91% at 2 years. Twenty-six patients received MR-guided SBRT for 31 liver metastases, 16% with online adaptation [22]. However, median follow-up was shorter than in our study (17 months vs. 22 months). The fact that four out of five local recurrences in our study occurred between 21 and 22 months after SBRT might explain the formally higher estimated local control rate in the described publication. Nonetheless, despite high local control in general, OS and especially PFS were rather low in both studies and underline the necessity for improved systemic therapy (respectively, 42% and 9% vs. 57% and 5% in our study). Moreover, every patient with primary pancreatic cancer in our study developed new liver metastases within 7 months after SBRT (Figure 3B), which should be kept in mind when discussing and evaluating SBRT for liver metastases. Drug resistance and high rates of adverse events pose a major challenge in the long-term treatment of systemic tumor diseases. Nonetheless, current developments in immunotherapy (including anti-PD1/PDL1- and CAR-T cell therapy) are promising [31,32].

Clinical utilization of online plan adaptation varies among radiotherapy departments between 16 and 100%, with some centers only performing adaptation in case of direct proximity to OARs [20,22,24]. Given our in-house experience with significant improvements in PTV coverage and OAR sparing, we always calculate an adapted plan if any constraint is compromised regardless of the distance to OARs, which is nearly always the case [27]. With a utilization rate of 56% in our study, plan adaptation rate seems to be rather low. However, this is due to the circumstance that plan adaptation began in February 2020, when it was clinically implemented in our department.

We demonstrated a BED >100 Gy to be a significant factor for superior local control (Figure 3B). Due to small sample sizes so far, no data for comparison with MR-guided SBRT are available in the literature. Nonetheless, our results fall in line with earlier studies on non-MR-guided SBRT of liver metastases: Ohri et al. found a BED of 100 Gy or lower to be associated with a substantially decreased 3-year local control (93% vs. 65%) [33]. Despite the fact that our study includes the largest patient population to date, the main limitation is the limited size. Moreover, when investigating clinical outcomes of MR-guided SBRT, one might be led astray by selection bias: direct MR-guidance now allows the SBRT of liver metastases even with moving OARs in close proximity (Figure 1). Some lesions, which were deemed not amenable for SBRT in the past, can now be irradiated with a (reduced) dose, tailored to the respective OARs. This might even lead to seemingly inferior local control rates when MR-guidance data further matures in the future. Thus, non-randomized trials like our presented study need to be interpreted with caution when compared with non-MR-guided SBRT. The ongoing MAESTRO trial in our department addresses this bias by comparing the benefit of online adapted MR-guided SBRT liver metastases with CBCT-guided SBRT [34]. If high-dose SBRT is feasible (≥100 Gy BED; alpha/beta = 10 Gy), patients are randomized between MR-guidance and CBCT-guidance. If not, patients receive upfront MR-guided SBRT to evaluate the highest possible dose of SBRT through MR-guidance. Similarly, the French phase-II RASTAF study assesses MR-guided dose escalation: If the liver metastasis is located far away from OARs, an additional fraction of 10 Gy is performed, with a total dose of 60 Gy instead of 50 Gy [35].

## 5. Conclusions

To our knowledge, we present the largest cohort of MR-guided liver SBRT, in particular in terms of online plan adaptation. Long-term local control was promising, with only mild acute toxicity. We confirm that high biological doses (BED10 Gy > 100 Gy) should be aimed for wherever possible. Nonetheless, systemic disease control remains challenging and patients need to be chosen carefully. Future randomized studies will assess the unbiased potential of MR-guided SBRT. 

## Figures and Tables

**Table 1:** Dose Constraints.

	3 fx	5 fx	8 fx	10 fx
esophagus 0.5 cc	<25.2 Gy	<34 Gy	<40 Gy	<43.5 Gy
stomach/intestine 0.5 cc	<22.2 Gy	<35 Gy	<40 Gy	<43.5 Gy
liver minus CTV ≥ 700 cc	<19.2 Gy	<24 Gy	<29 Gy	<32 Gy
kidney mean dose	<8.5 Gy	<10 Gy	<11.5 Gy	<12 Gy
spinal cord 0.1 cc	<21.6 Gy	<27 Gy	<32 Gy	<35 Gy
heart 0.5 cc	<26 Gy	<29 Gy	<60 Gy	<66 Gy
All dose constraints were (strict) minimum requirements. Whenever possible, lower thresholds were aimed for.				

**Table 2:** Patient Characteristics (n = 40).

median age	62 years (37–89 years)
median Karnofsky Score	90% (70–100%)
female/male	19/21
prior liver surgery	13 (33%)
prior liver microwave ablation	1 (3%)
prior liver radiotherapy	4 (10%)
systemic therapy within 4 weeks before irradiation	27 (68%)
systemic therapy within 4 weeks after irradiation	16 (40%)
metastatic disease in prior medical history *	29 (73%)
liver metastases in prior medical history *	26 (65%)
>3 liver metastases in prior medical history *	17 (43%)
total number of irradiated liver targets per patient	
n = 1	35 (88%)
n = 2	3 (7%)
n = 3	0
n = 4	2 (5%)

* excluding the irradiated liver metastases.

**Table 3:** Irradiation treatment characteristics (n = 54 lesions).

Primary Tumor	
Colorectal carcinoma	21 (39%)
Breast cancer	10 (19%)
Pancreatic cancer	9 (17%)
Esophageal cancer	3 (6%)
Melanoma	3 (6%)
Other *	8 (15%)
median PTV volume	54 cc (10–445 cc)
median prescribed total dose	50 Gy (45–60 Gy)
median number of fractions	5 (3–12)
median EQD2 (α/β = 10)	83 Gy (54–94 Gy)
median BED (α/β = 10)	100 Gy (65–113 Gy)
Clinical events (during and 8 weeks after irradiation)	
Grade 1	14 (26%)
Grade 2	4 (7%)
Grade ≥ 3	0

BED: biologically effective dose; EQD2 = equivalent dose at 2 Gy; PTV: planning target volume; * each n = 1 adenoid cystic/cholangiocellular/papillary/parotic gland carcinoma, non-small cell lung/prostate/renal/bladder cancer.

## Data Availability

The data presented in this study are available on request from the corresponding author.
